# P-496. Adolescent Provider Perspectives on Sexual Health Screening and Preventive Care

**DOI:** 10.1093/ofid/ofaf695.711

**Published:** 2026-01-11

**Authors:** Nicholas Venturelli, Douglas Krakower, Carly Guss

**Affiliations:** Boston Children's Hospital, Boston, MA; Harvard Medical School, Boston, MA; Boston Children's Hospital, Boston, MA

## Abstract

**Background:**

Youth ages 13–24 account for nearly 20% of new HIV diagnoses in the U.S., yet rates of HIV screening and PrEP prescribing remain low. Primary care providers (PCPs) play a key role in prevention but face unique barriers when caring for adolescents. We aimed to evaluate knowledge, attitudes, practices, and perceived barriers to adolescent sexually transmitted infection (STI) screening and HIV prevention counseling among U.S. primary care providers.

**Figure:**
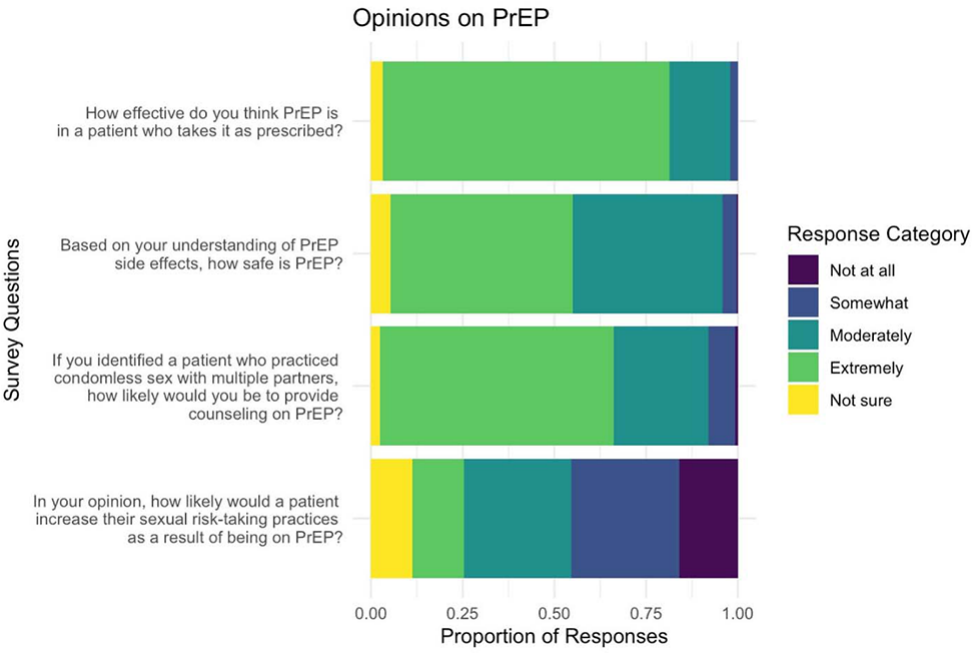

**Figure:**
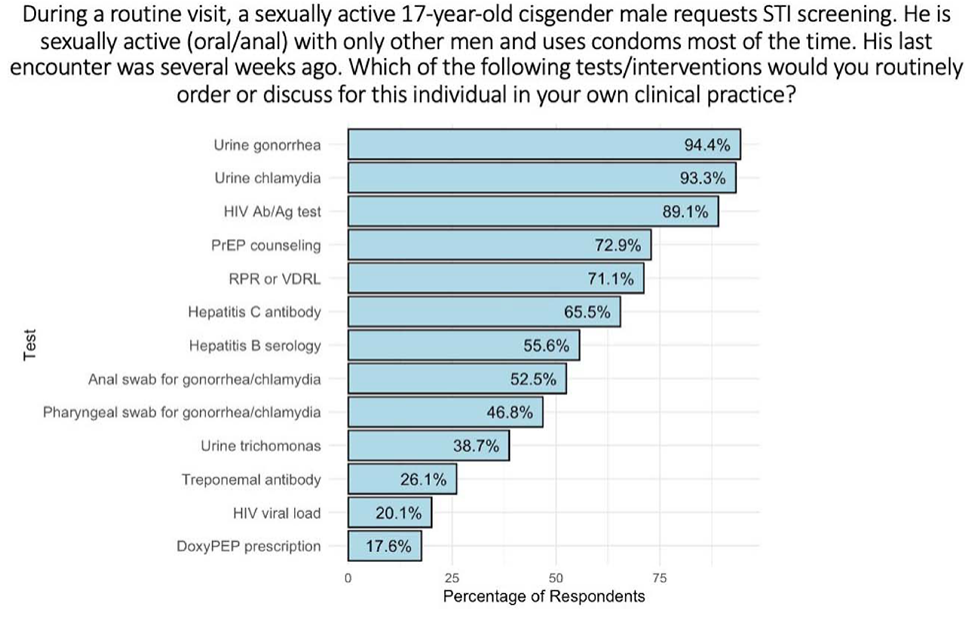

**Methods:**

We conducted a national cross-sectional survey of adolescent-serving PCPs using the Qualtrics platform. Eligible participants included physicians, nurse practitioners, and physician assistants from a range of specialties. The survey captured provider demographics and measured four domains: (1) knowledge of PrEP and STI screening guidelines, (2) attitudes and perceived barriers to STI prevention, (3) current practices, and (4) interest in implementation strategies. Responses were analyzed descriptively and compared across provider types using Kruskal-Wallis tests.

**Figure:**
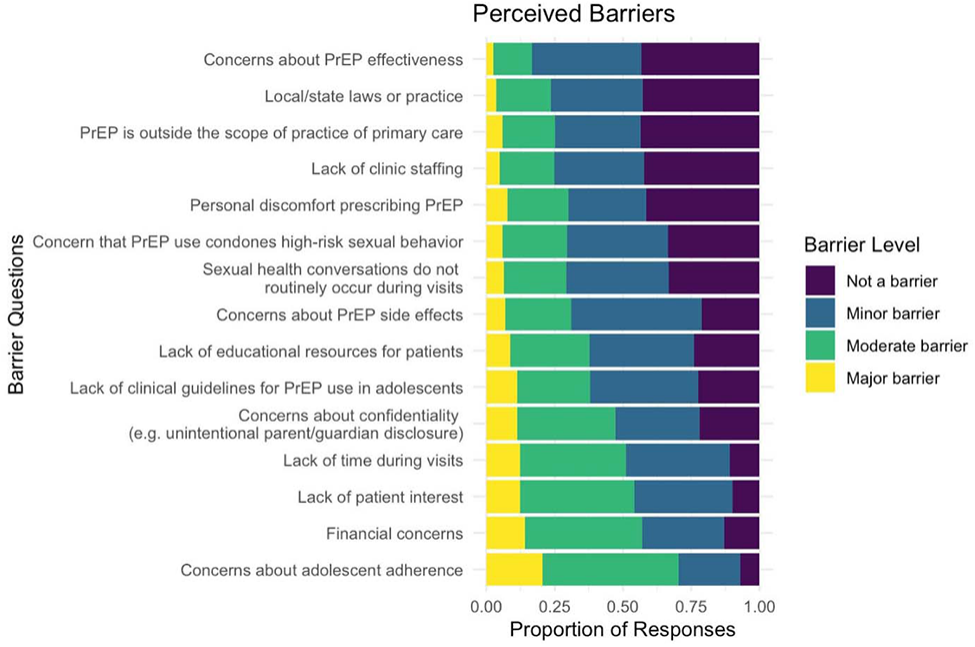

**Results:**

We received 284 responses from a diverse national sample: 57% Family Medicine, 16% Pediatrics, and 11% Internal Medicine. Most (90%) reported comfort discussing sexual health with adolescents, and 85% supported universal HIV screening. While 88% of providers stated they were “moderately” or “extremely” likely to counsel on PrEP in a patient who qualified (Fig. 1), when later presented with a clinical scenario, only 73% of providers recommended PrEP counseling, with fewer than half sending a pharyngeal/anal swab for chlamydia or gonorrhea (Fig. 2). Relatedly, 42% of pediatricians had never initiated a PrEP conversation in their practice, compared to 16% of family and 19% of internal medicine providers (p < 0.001). Key barriers for sexual health preventative care included time constraints, confidentiality concerns, adherence worries, and perceived patient disinterest. Fewer cited moral or legal objections (Fig. 3). High-priority interventions included EMR-based order sets and predictive algorithms.

**Conclusion:**

Despite strong support for HIV prevention, substantial gaps exist in actual STI and PrEP knowledge and implementation—especially among pediatricians. Systems-level interventions could address key barriers and expand PrEP delivery.

**Disclosures:**

All Authors: No reported disclosures

